# Potential targets of transforming growth factor-beta1 during inhibition of oocyte maturation in zebrafish

**DOI:** 10.1186/1477-7827-3-53

**Published:** 2005-09-30

**Authors:** Gurneet Kohli, Eric Clelland, Chun Peng

**Affiliations:** 1 Department Of Biology, York University, Toronto, Ontario, M3J 1P3, Canada

## Abstract

**Background:**

TGF-beta is a multifunctional growth factor involved in regulating a variety of cellular activities. Unlike mammals, the function of TGF-beta in the reproduction of lower vertebrates, such as fish, is not clear. Recently, we showed that TGF-beta1 inhibits gonadotropin- and 17alpha, 20beta-dihydroxyprogesterone (DHP)-induced maturation in zebrafish. The aim of the present study was to investigate the mechanisms underlying this action.

**Method:**

To determine if the effect of TGF-beta1 on oocyte maturation involves transcription and/or translation, ovarian follicles were pre-treated with actinomycin D, a blocker of transcription, and cyclohexamide, an inhibitor of translation, and incubated with hCG or DHP, either alone or in combination with TGF-beta1 and oocyte maturation scored. To determine the effect of TGF-beta1 on mRNA levels of several key effectors of oocyte maturation, three sets of experiments were performed. First, follicles were treated with control medium or TGF-beta1 for 2, 6, 12, and 24 h. Second, follicles were treated with different concentrations of TGF-beta1 (0 to 10 ng/ml) for 18 h. Third, follicles were incubated with hCG in the absence or presence of TGF-beta1 for 18 h. At the end of each experiment, total RNA was extracted and reverse transcribed. PCR using primers specific for 20beta-hydroxysteroid dehydrogenase (20beta-HSD) which is involved in DHP production, follicle stimulating hormone receptor (FSHR), luteinizing hormone receptor (LHR), the two forms of membrane progestin receptor: mPR-alpha and mPR-beta, as well as GAPDH (control), were performed.

**Results:**

Treatment with actinomycin D, a blocker of transcription, reduced the inhibitory effect of TGF-beta1 on DHP-induced oocyte maturation, indicating that the inhibitory action of TGF-beta1 is in part due to regulation of gene transcription. Treatment with TGF-beta1 caused a dose and time-dependent decrease in mRNA levels of 20beta-HSD, LHR and mPR-beta in follicles. On the other hand, TGF-beta1 had no effect on mPR-alpha mRNA expression and increased FSHR mRNA levels. Furthermore, hCG upregulated 20beta-HSD, LHR and mPR-beta mRNA levels, but this stimulatory effect was blocked by TGF-beta1.

**Conclusion:**

These findings suggest that TGF-beta1 acts at multiple sites, including LHR, 20beta-HSD and mPR-beta, to inhibit zebrafish oocyte maturation.

## Background

Transforming Growth Factor-β1 (TGF-β1) is the prototypical member of the TGF-β family [1,2]. Members of this family are implicated in diverse physiological processes, including reproduction. Three isoforms of TGF-β (TGF-β1, -β2, and -β3) are expressed in the mammalian ovary [2-4]. They have been shown to regulate follicle development, steroidogenesis, oocyte maturation, ovulation and follicular atresia [2-4]. There is molecular evidence for the presence of TGF-β1–3 in fish [5-7]. However, the role of TGF-β in fish reproduction is not well understood. Studies in zebrafish have suggested that TGF-β inhibits oocyte maturation [8]. In the goldfish, TGF-β has been reported to inhibit ovarian steroid production [9].

Ovarian development in fish is broadly divided into two major phases: growth and maturation. During oocyte growth, follicle stimulating hormone (FSH) stimulates production of estradiol-17β from the ovary. Estradiol-17β stimulates the production of vitellogenin by the liver. Vitellogenin is taken up by the developing oocyte and cleaved to yolk protein, which serves as a nutritional reserve for the developing embryo [8,10,11]. Oocyte maturation in teleosts is triggered by the release of leutinizing hormone (LH) from the pituitary. LH stimulates a number of signaling cascades culminating in the production of 17α-hydroxyprogesterone (17α-HP). In the granulosa cells, under the action of 20β-hydroxysteroid dehydrogenase (20β-HSD), 17α-HP is converted to 17α, 20β-dihydroxyprogesterone (DHP), the maturation inducing hormone (MIH) in cyprinids, such as zebrafish and goldfish. MIH activates the cytoplasmic maturation promoting factor (MPF), which is made up of two subunits: cyclin B (a regulatory subunit) and cdc2 (a catalytic subunit). MIH stimulates the *de novo *synthesis of cyclin B. Cyclin B protein binds to cdc2 to form MPF. The newly formed MPF is activated by phosphorylation of cdc2 on threonine 161. The active MPF, then, stimulates all the changes associated with oocyte maturation, such as germinal vesicle break down (GVBD), spindle formation, chromosome condensation and allows the transition from G2/M phase of meiosis [12-15].

Two isoforms of the MIH receptor, designated as membrane progestin receptor-α (mPR-α) and mPR-β, have recently been cloned in zebrafish [16]. Microinjection of zebrafish oocytes with antisense oligonucleotides to either mPR-α or mPR-β or both receptors has been shown to block MIH-induced maturation, indicating that both play a role in zebrafish oocyte maturation [17]. Originally discovered in sea-trout oocytes, several isoforms of mPR have also been discovered in humans and other vertebrates [16-20].

The zebrafish model has been used extensively for studies on early embryonic development. This model is also very useful for the investigation of ovarian follicle development and maturation because the zebrafish ovary contains ovarian follicles at different stages of development. We and others have been using zebrafish to examine the role of TGF-β superfamily in oocyte maturation [8,21-23]. Our previous work has shown that TGF-β1 inhibits human chorionic gonadotropin (hCG) and MIH-induced maturation in zebrafish [8]. We have also observed a decrease in TGF-β1 mRNA expression in maturing follicles. These findings suggest that TGF-β1 may play a role in preventing premature oocyte maturation in zebrafish. The present study is an attempt to elucidate the mechanisms underlying the inhibitory effect of TGF-β1 by identifying the potential target genes of TGF-β1. We report here our recent findings on the effect of TGF-β1 on the important effectors of oocyte maturation in zebrafish, including: 20β-HSD, LHR, mPR-α, and mPR-β.

## Materials and methods

### Animals

Adult zebrafish, *Danio rerio*, were purchased from a local pet supplier (Fish & Bird Emporium, Brampton, ON) and maintained in 10 L tanks of an AHAB System(Aquatic Habitats, FL) at 26°C, under a 14-h light, 10-h dark photoperiod. The fish were fed twice daily with tropical fish food and supplemented with freshly hatched brine shrimp two or three times a week. Experiments were performed according to the *Guide to the Care and Use of Experimental Animals *published by the Canadian Council on Animal Care.

### *In vitro *culture of zebrafish follicles

Female zebrafish that have a full-grown ovary were anesthetized using 3-aminobenzoicacid ethyl ester (Sigma-Aldrich Canada Inc., Oakville, ON) and decapitated. The ovaries were removed and follicles greater than 0.52 mm in diameter were collected, since previous studies in the zebrafish have shown that only follicles of this size can undergo maturation *in vitro *in response to hormones [21]. Approximately 20 follicles were placed into each well of a 24-well culture plate and pre-treated at 26°C in either 1 ml of modified Cortland's medium (MCM) or MCM + chemicals such as Actinomycin D or Cyclohexamide (Sigma-Aldrich, Mississauga, ON) for 2 hours. Pre-treated follicles were then incubated with the control medium, human recombinant TGF-β1 (R&D Systems, Minneapolis, MN), hCG (kindly provided by Dr. A. F. Parlow, National Hormone and Peptide Program, Torrance, CA), MIH (17α, 20β-DHP;Sigma-Aldrich Canada), either alone or in combination, as previously described [8]. Maturation was scored after 18 hours of incubation. Follicles that underwent germinal vesicle breakdown (GVBD) could be identified by their acquired translucency.

### Total RNA extraction

Approximately 80 follicles per treatment group were used for RNA extraction. Total RNA was extracted using an RNeasy Mini kit (Qiagen Inc., Mississauga, ON) according to the manufacturer's suggested protocol. The RNase-free deoxyribonuclease Set (Qiagen) was also used during RNA isolation to remove any potential genomic DNA contamination.

### Reverse Transcription (RT) and polymerase chain reaction (PCR)

Five micrograms of total RNA were reverse transcribed to cDNA at 37°C for 1.5 h in a total volume of 50 μl as previously described [21]. PCR was carried out in the presence of 10 mM Tris-HCl (pH 8.3), 50 mM KCl, 2.0 mM MgCl_2_, 50 μM deoxynucleotide triphosphate, 1U Hotstar Taq (Qiagen) and 5 pmol primers. The primers for 20β-HSD, FSHR, LHR, mPR-α and mPR-β were designed according to the sequences from GenBank (Table 1). The semi-quantitative RT-PCR was validated by peforming PCR reactions for different cycles to determine the cycle number that generated half maximal PCR product for each gene studied. PCR was carried out on an Eppendorf Master Cycler (Eppendorf AG, Hamburg, Germany) with the cycling profile: 20 s at 94°C, 30 s at 60–65°C (depending on the primer sets used), 30 s at 72°C followed by a 7-minute final extension at 72°C. The PCR products were electrophoresed on 1.5% agarose gels and visualized by ethidium bromide staining. The spot density of each PCR product was determined using the Fluorchem v2.0 Software (Alpha Innotech Corporation, San Leandro, CA). GAPDH was used as an internal control to normalize the variation in mRNA concentration in the RT reaction. The mRNA level of each gene was first normalized with the GAPDH level and then expressed as percent of the control.

**Table 1:** List of primers and their sequences

Gene	Sequence (5'-3')	Accession #
20β-HSD	Forward: TGC ACG AGT GGT CAA TGT GTC Reverse: ACT AGC TGT CCA TGC GGC TCT	AF298898
FSHR	Forward: GGA TTC TTC ACC GTC TTC TCC Reverse: TGT AGC TGC TCA ACT CAA ACA	AY278107
LHR	Forward: GGC GAA GGC TAG ATG GCA CAT Reverse: TCG CCA TCT GGT TCA TCA ATA	AY424302
mPR-α	Forward: CAG CGC CTA CTT CTT CTC GT Reverse: CAC TGC ATC ATG AGC CAA AT	AY149121
mPR-β	Forward: ACA ACG AGC TGC TGA ATG TG Reverse: ATG GGC CAG TTC AGA GTG AG	AY14920

### Statistical Analysis

All values are expressed as mean ± SEM of 3–4 replicates in one representative experiment. All experiments were performed three times to confirm the results using different batches of animals. To determine the statistical difference among different groups at the same time point, multiple group comparisons were performed by one-way ANOVA, followed by a Student-Newman-Keuls multiple group comparisons test, using the GraphPad InStat Software (GraphPad Inc., San Diego, CA). *P *< 0.05 was considered significant.

## Results

### Effects of transcriptional and translational inhibitors on final oocyte maturation

**Figure 1:**
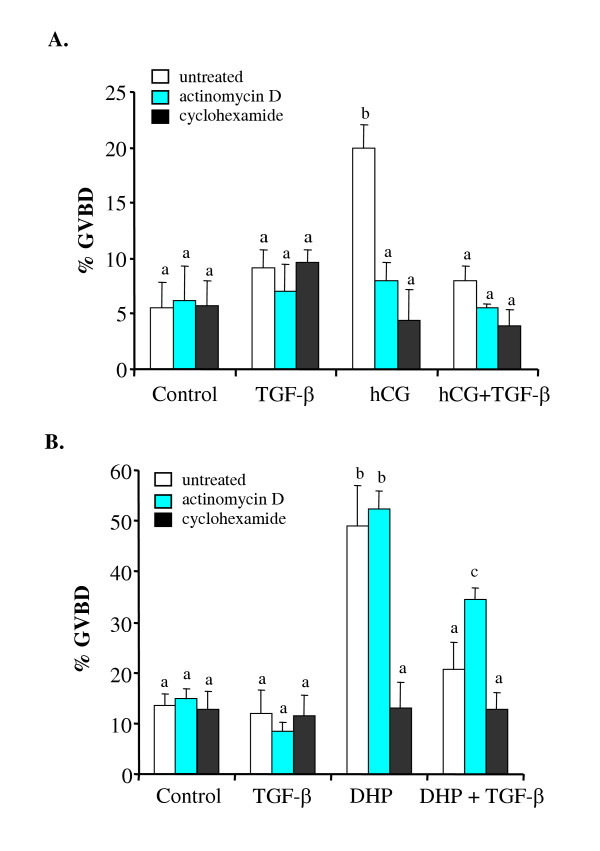
Effects of transcriptional and translational inhibitors on oocyte maturation. Ovarian follicles were pre-treated for 2 hours with 1 mg/ml Actinomycin D, Cyclohexamide or medium (untreated) and then incubated with: (A) Medium only (control), TGF-β1 (10 ng/ml), hCG (100 IU/ml), or a combination of hCG and TGF-β1 or (B) Medium only (control), TGF-β1 (10 ng/ ml), MIH (100 ng/ml) or a combination of MIH and TGF-β1 for 18 hours. The rate of maturation was scored as percentage of follicles that underwent GVBD. Data represent the mean ± SEM of one representative experiment with four replicates. *Different *letters denote statistical significance (*P *< 0.05).

To determine if the effect of TGF-β on oocyte maturation requires transcription and/or translation, follicles were pretreated with cyclohexamide, an inhibitor of translation, or actinomycin D, an inhibitor of transcription. Pretreatment with cyclohexamide completely blocked hCG- and MIH-induced oocyte maturation. Pretreatment with actinomycin D blocked hCG-, but not MIH-, induced oocyte maturation (Fig. 1A). Interestingly, the inhibitory effect of TGF-β1 on MIH-induced maturation was partially reversed in the presence of actinomycin D (Fig. 1B), suggesting that the inhibitory effect of TGF-β1 on oocyte maturation involves in part transcriptional regulation.

### Semi-quantitative RT-PCR Validation

**Figure 2:**
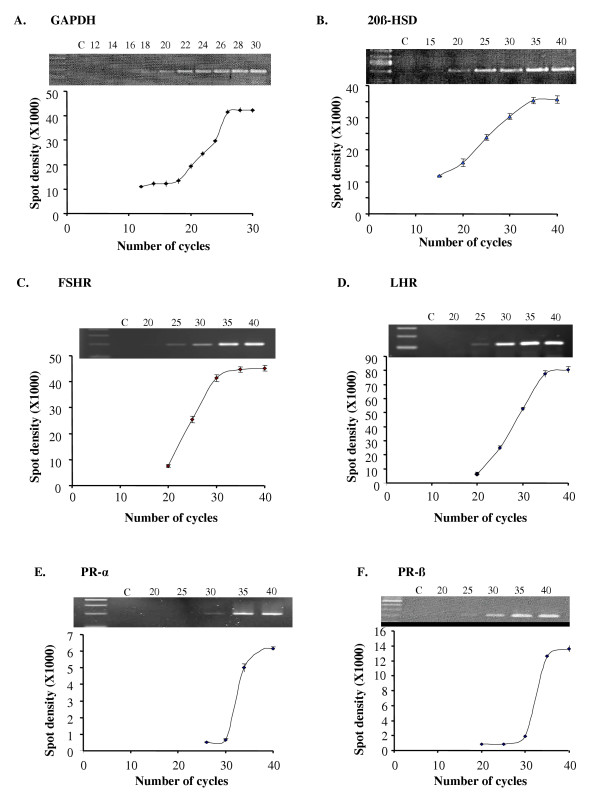
Validation of semi-quantitative RT-PCR for GAPDH (A), 20β-HSD (B), FSHR (C), LHR (D), mPR-α (E) and mPR-β (F). PCRs were performed using zebrafish ovarian cDNA as the template, amplified for varying cycle numbers and the density of the PCR products was quantified. Each value represents the mean ± SEM of three replicates in one representative RT-PCR. Representative ethidium bromide stained gel pictures were included. C = negative control; number on each lane represents the number of PCR cycles performed.

Semi-quantitative RT-PCR assays were developed to examine the effect of TGF-β1 on the mRNA expression of 20β-HSD, FSHR, LHR, mPR-α and mPR-β. PCR assays were performed using ovarian cDNA as template for varying cycle numbers and spot densities of the resulting products were measured. A cycle number within the exponential phase of the amplification curve was chosen for quantifying the expression of each gene in subsequent experiments. Accordingly, 20, 23, 30, 31, 33 and 32 cycles of PCR for GAPDH, 20β-HSD, FSHR, LHR, mPR-α and mPR-β, respectively, were selected as the optimal cycle numbers for measuring the levels of mRNA expression (Fig. 2).

### Effect of TGF-β1 on the mRNA expression of 20β-Hydroxysteroid dehydrogenase

**Figure 3:**
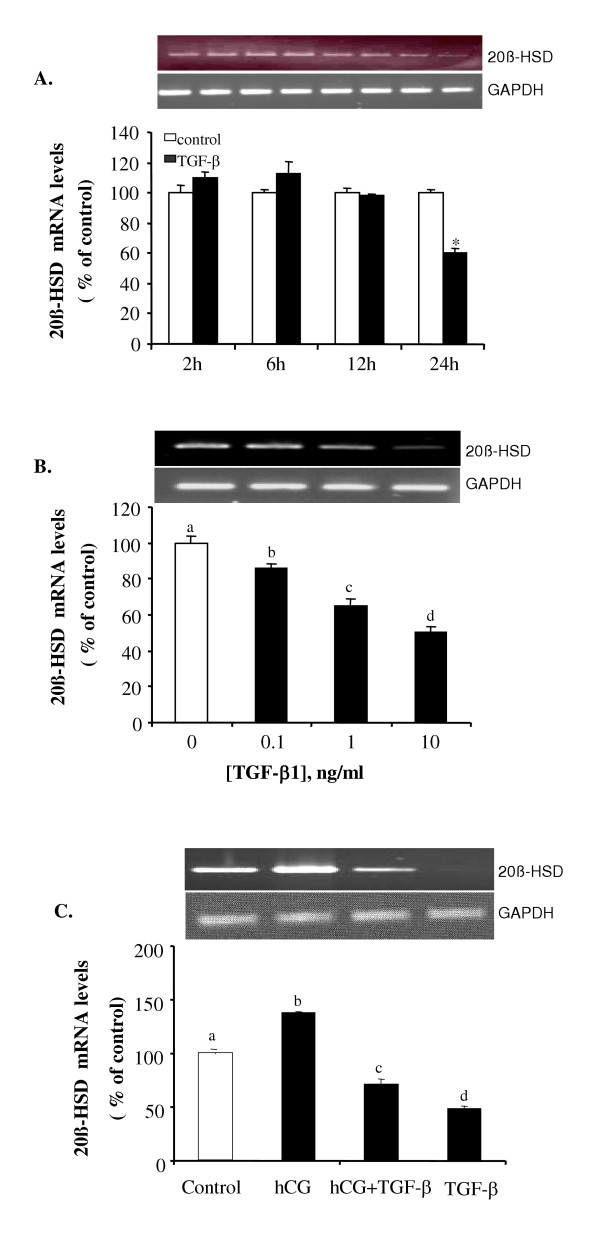
TGF-β1 inhibits mRNA expression of 20β-HSD. (A) Follicles were treated with control medium or 10 ng/ml of TGF-β1 for 2, 6, 12 and 24 hours. (B) Follicles were treated with different concentrations (0, 0.1,1 and 10 ng/ml) of TGF-β1 for 18 hours. (C) Follicles were treated with control medium, hCG (100 IU/ml), TGF-β1 (10 ng/ml), or hCG+ TGF-β1 for 18 hours. Total RNA was extracted and subjected to RT-PCR using primers for 20β-HSD and GAPDH. Each value represents the mean ± SEM of three replicates in one representative RT-PCR reaction. 20β-HSD mRNA levels are expressed as percent of control after normalized with the GAPHD levels. Different letters above the bars denote statistical significance (P < 0.05). *, *P *< 0.05 vs. control. The insets show representative ethidium bromide stained gels. GAPDH gels are the same for Figs. 4-7.

To test whether TGF-β1 affects the mRNA expression of 20β-HSD, a time course study was performed where follicles were treated with 10 ng/ml of TGF-β1 for 2, 6, 12 and 24 hours. A set of untreated follicles was included for each time point as controls. Total RNA was extracted and RT-PCRs were performed. A time-dependant decrease in 20β-HSD mRNA expression relative to control levels was found in TGF-β1 treated follicles. A significant inhibitory effect was observed at 24 hours of treatment (Fig. 3A). A dose-response study was performed where follicles were treated with increasing doses of TGF-β1 (0, 0.1, 1 and 10 ng/ml) for 18 hours. Total RNA was extracted from each set of treated follicles and subjected to RT-PCR analyses. A dose-dependant decrease in 20β-HSD expression in response to TGF-β1 treatment was found (Fig. 3B). The strongest inhibition was seen upon treatment with 10 ng/ml of TGF-β1. Finally, the combinational effect of hCG and TGF-β1 on 20β-HSD mRNA expression was tested. Follicles were incubated with control medium, hCG (100 IU/ml), TGF-β1 (10 ng/ml), or hCG plus TGF-β1, for 18 hours. Treatment with hCG resulted in an increase in 20β-HSD mRNA levels. However, treatment with TGF-β1 decreased basal and hCG-induced 20β-HSD mRNA levels (Fig. 3C).

### Effect of TGF-β1 on the mRNA expression of follicle stimulating hormone receptor

**Figure 4:**
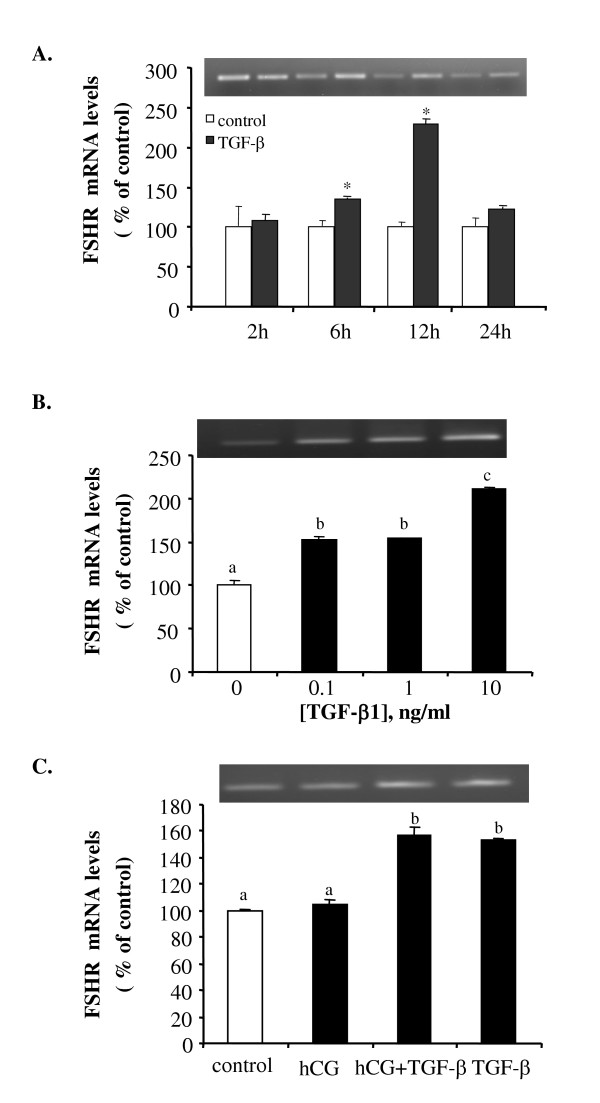
TGF-β1 stimulates FSHR mRNA expression. (A) Follicles were incubated with control medium or 10 ng/ml of TGF-β1 for 2, 6, 12 and 24 hours. (B) Follicles were treated with different concentrations (0, 0.1,1 and 10 ng/ml) of TGF-β1 for 18 hours. (C) Follicles were treated with medium (control), hCG (100 IU/ml), TGF-β1 (10 ng/ml), or a combination of hCG and TGF-β1 for 18 hours. At the end of each incubation, total RNA was extracted and reverse transcribed. PCR was carried out using primers for FSHR and GAPDH. Each value represents the mean ± SEM of three replicates in one representative RT-PCR. Statistical significance (P < 0.05) is indicated by either an * or a different letter. The insets show the representative ethidium bromide stained gels.

Treatment with TGF-β1 resulted in a significant increase in FSHR mRNA levels from 6 to 18 hours after treatment, with the maximal stimulation at 12 hours post treatment (Fig. 4A, B). The stimulatory effect of TGF-β1 on FSHR mRNA expression was observed for all doses tested (Fig. 4B). Treatment with hCG had no significant effect on basal FSHR mRNA expression when compared to the control (Fig. 4C). Similarly, hCG did not affect TGF-β1-induced FSHR mRNA levels (Fig. 4C).

### Effect of TGF-β1 on the mRNA expression of the luteinizing hormone receptor

**Figure 5:**
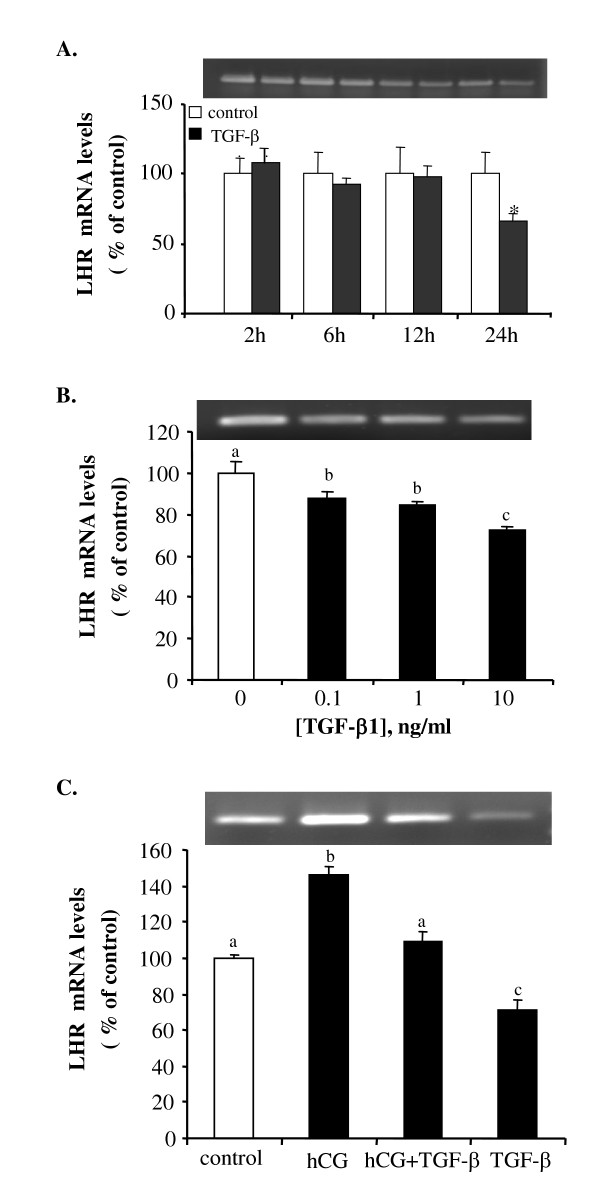
TGF-β1 suppresses LHR mRNA expression. Follicles were treated with (A) 10 ng/ml of TGF-β1 for 2, 6, 12 and 24 hours; (B) 0.1, 1, or 10 ng/ml of TGF-β1 for 18 hours; and (C) hCG (100 IU/ml), TGF-β1 (10 ng/ml), either alone or in combination, for 18 hours. Each value represents the mean ± SEM of three replicates in one representative RT-PCR. Different letters denote statistical significance (*P *< 0.05). The insets show the original ethidium bromide stained gels.

TGF-β1 significantly decreased LHR mRNA expression at 18 and 24 hours after treatment, but had no effect at 2 to 12 hours post treatment (Fig. 5A, B). At 18 hours after treatment, TGF-β1 inhibited LHR mRNA expression in a dose-dependant manner (Fig. 5B). Treatment with hCG increased LHR mRNA levels. However, when TGF-β1 was added together with hCG, the stimulatory effect of hCG on LHR mRNA expression was blocked (Fig. 5C).

### Effect of TGF-β1 on the mRNA expression of membrane progestin receptors

**Figure 6:**
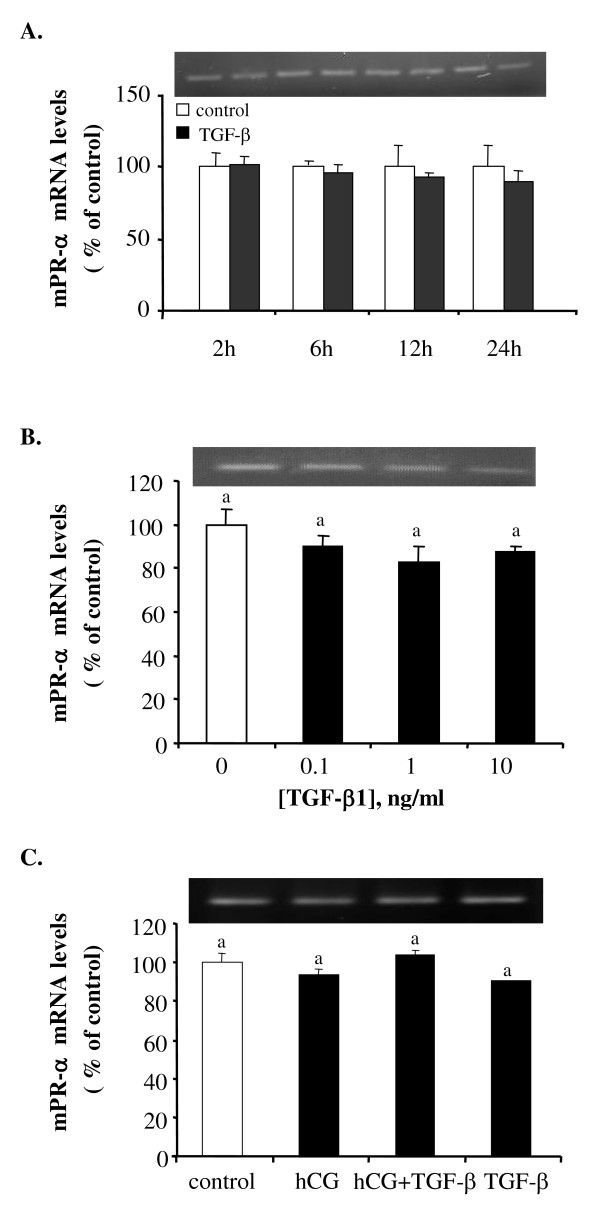
TGF-β1 has no effect on mPR-α mRNA expression. Follicles were incubated with (A) 10 ng/ml of TGF-β1 for 2, 6, 12 and 24 hours; (B) different concentrations of TGF-β1 for 18 hours; and (C) medium only (control), hCG (100 IU/ml), TGF-β1 (10 ng/ml) or hCG + TGF-β1 for 18 hours. Each value represents the mean ± SEM of three replicates in one representative RT-PCR. The insets show representative ethidium bromide stained gels. Neither hCG nor TGF-β1 had an effect on mPR-α mRNA expression.

**Figure 7:**
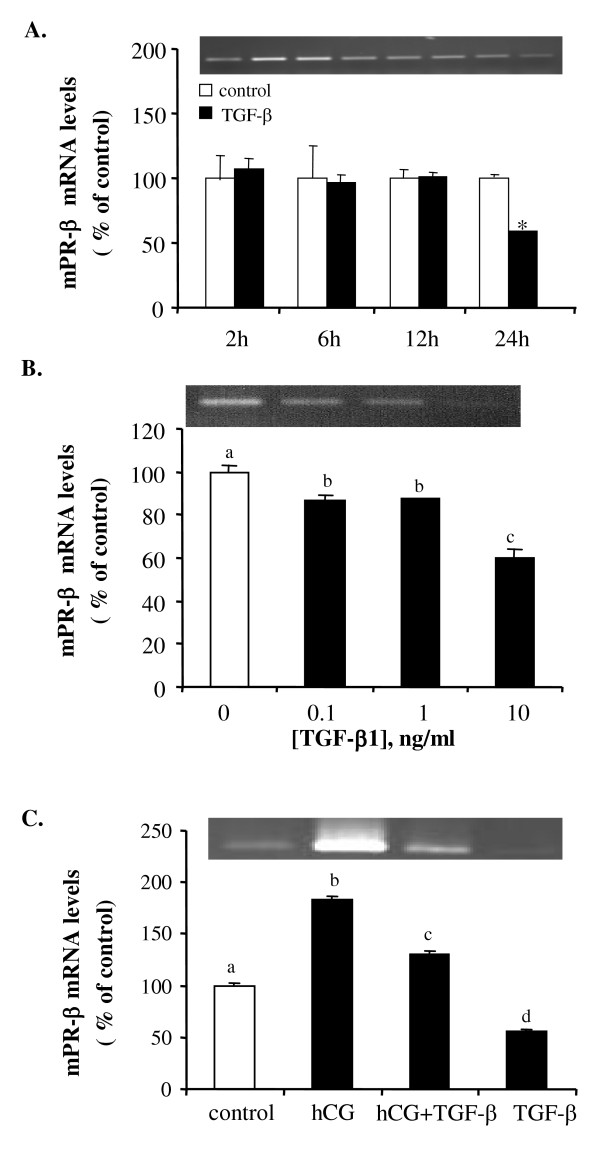
TGF-β1 downregulates mPR-β mRNA expression. (A) Time course study of the effect of TGF-β1 on mPR-β mRNA expression. Follicles were treated with 10 ng/ml of TGF-β1 for 2, 6, 12 and 24 hours. (B) Follicles were treated with different concentrations (0, 0.1,1 and 10 ng/ml) of TGF-β1 for 18 hours. (C) Follicles were incubated with medium (control), hCG (100 IU/ml), TGF-β1 (10 ng/ml) or hCG + TGF-β1 for 18 hours. Each value represents the mean ± SEM of three replicates in one representative RT-PCR. Different letters or "*" denote statistical significance (*P *< 0.05). The insets show the representative ethidium bromide stained gels.

No significant effect of TGF-β1 on mPR-α mRNA expression was observed. The time course study showed no significant difference in the mRNA expression of mPR-α at any of the time points examined (Fig. 6A). Similarly, treatment with different concentrations of TGF-β1 for 18 hours did not result in a significant change in mPR-α mRNA levels (Fig. 6B). Neither hCG nor TGF-β1 had a significant effect on mPR-α mRNA expression (Fig. 6C). In contrast to mPR-β, similar treatment with TGF-β1 resulted in a dose- and time-dependent inhibition of mPR-β mRNA levels. The inhibitory effect was observed at 18 and 24 hours after treatment and all doses of TGF-β1 tested caused a significant decrease in mPR-β mRNA levels (Fig. 7A, B). Incubation of follicles with hCG resulted in a strong stimulation of mPR-β mRNA expression. The stimulatory effect of hCG on mPR-β mRNA expression of was partially blocked by TGF-β1 (Fig. 7C).

## Discussion

Currently, little is known about the role of TGF-β in regulating ovarian functions in lower vertebrates, such as fish. Recent studies have suggested that TGF-β inhibits steroid production in the goldfish ovary [9] and oocyte maturation in zebrafish [8]. In the present study, we further examined the cellular mechanisms underlying the inhibitory effect of TGF-β in oocyte maturation. We have shown that TGF-β1 inhibits mRNA expression of 20β-HSD, the key enzyme involved in MIH production, as well as LHR and mPR-β. These novel findings suggest that TGF-β1 inhibits multiple targets in the oocyte maturation pathway, both upstream and downstream of MIH.

One of the potential targets of TGF-β1 identified in this study is 20β-HSD. We found that TGF-β1 inhibited basal and hCG-induced 20β-HSD mRNA levels. TGF-β1 alone induced a dose- and time-dependent inhibitory effect on 20β-HSD mRNA expression. Treatment with hCG increased 20β-HSD mRNA levels; however, in the presence of TGF-β1, the effect of hCG was blocked. 20β-HSD activity in the ovary and its stimulation by gonadotropins and cAMP-enhancing drugs has been demonstrated in many species [10,11,24-28]. Our finding that hCG stimulates 20β-HSD is consistent with studies in mammals [24,28] and a recent study in Nile Tilapia, which reported an increase in the mRNA expression of 20β-HSD in response to hCG treatment [26]. The observation of decreased 20β-HSD mRNA levels after TGF-β1 treatment suggests that TGF-β1 may inhibit 20β-HSD activity, leading to a decrease in MIH production. This notion is supported by a recent report that TGF-β1 inhibits the conversion of 17α-HP to DHP in the goldfish [9]. It is possible that one of the actions of TGF-β1 is to decreases MIH production, and thus inhibits oocyte maturation.

In this study, we observed that hCG increased LHR but had no effect on FSHR mRNA levels. Several studies conducted in fish on the effect of gonadotropin on LHR and FSHR expression have yielded inconsistent results. In African and Channel catfish, it has been reported that hCG treatment caused an activation of the LHR and cAMP mediated pathways, as well as a slight increase in FSHR mRNA levels [29-31]. However, a two-fold increase in FSHR expression, but no change in LHR expression in pre-maturational follicles of rainbow trout in response to treatment with partially purified gonadotropins has also been reported [32]. The reason for such discrepancy is unclear, however, it could be due to species specificity or variation in treatment conditions.

An interesting finding from this study is that TGF-β1 differentially regulates FSHR and LHR mRNA levels. We observed that basal FSH receptor mRNA levels were increased, while basal- and hCG-induced LH receptor mRNA levels were decreased, upon incubation of follicles with TGF-β1. TGF-β1 has been reported to regulate LH and FSH binding sites in mammals, however, whether TGF-β1 has a stimulatory or inhibitory effect appears to be species-dependant. TGF-β1 decreased basal and FSH-induced LH binding sites in porcine granulosa cells but enhanced FSH-induced LH binding in rat granulosa cells [33]. FSH induced FSH binding in porcine granulosa cells and this effect was attenuated by TGF-β1. On the other hand, FSH decreased its own binding levels in rat granulosa cells and this effect was also blocked by TGF-β1 [33]. Our finding that TGF-β1 increased FSHR mRNA levels is consistent with previous studies in rat granulosa cells [34,35]. However, our observation that TGF-β1 decreased LHR mRNA levels is opposite to studies in rat [36] and chicken [37] granulosa cells. In these studies, it was reported that TGF-β1 induced LHR mRNA expression. Whether this is due to species variation as in the case of rat and pig or to the difference in follicle development between fish and higher vertebrates awaits more studies in fish species. The finding that TGF-β1 inhibited LHR mRNA expression suggests that the inhibitory effect of TGF-β1 on hCG-induced oocyte maturation may be due, in part, to the downregulation of LH receptor by TGF-β1. Since FSH is known to be a major regulator of oocyte growth [10,11], by stimulating FSH receptor expression TGF-β1 may play a role in promoting follicle growth. These findings, together with our previous observation that TGF-β1 mRNA levels are higher in growing follicles than in maturing follicles, suggest that TGF-β1 may stimulate follicle development and inhibit precocious oocyte maturation in the zebrafish ovary. This possibility will be investigated further in the future.

A recent study has shown that microinjection of zebrafish oocytes with antisense oligonucleotides to either mPR-α or mPR-β or both causes similar marked decreases in the rates of oocyte maturation, suggesting that both subtypes are obligatory for oocyte maturation in zebrafish [17]. In this study, we observed a strong induction of mPR-β mRNA levels by hCG and an inhibitory effect of TGF-β1 on both basal and hCG-induced mPR-β mRNA expression. These findings support the role of mPR-β in oocyte maturation and suggest that one of the mechanisms by which TGF-β1 inhibits hCG- and MIH-induced oocyte maturation is by the downregulation of MIH receptors, specifically mPR-β. However, we found that neither hCG nor TGF-β1 had an effect of mPR-α mRNA levels, suggesting that the two membrane progestin receptors are under differential regulation. It has been reported that hCG caused an increase in the mPR protein expression in sea-trout oocytes [18]. The sea-trout mPR has a higher homology to mPR-α (80%) than to mPR-β (46%). It remains to be determined if hCG regulates mPR-α and mPR-β protein levels in the zebrafish. Recently, Kazeta et al. (2005) reported that hCG did not change mRNA levels of mPR-α and mPR-β at 5 and 10 h after hCG treatment [38]. The difference between this and our studies may be due to the duration of hCG treatment as 18 h was used in our study.

## Conclusion

Based on our findings that TGF-β1 downregulates basal and hCG-induced LHR, 20β-HSD, and mPR-β mRNA levels, we propose that TGF-β1 acts upon multiple targets to exert its inhibitory effect on oocyte maturation. TGF-β1 may downregulate LHR, leading to decreased signal transduction and decreased production of 17α-hydroxyprogesterone. TGF-β1 may inhibit basal and gonadotropin-induced MIH production by inhibiting 20β-HSD. Finally, TGF-β1 may inhibit the expression of the MIH receptor, such as mPR-β, on the oocyte surfaces, leading to a reduction of MPF activation and subsequent oocyte maturation. This model can be tested in the future by examining the protein levels on these molecules once antibodies become available. Similarly, the physiological significance of TGF-β on oocyte maturation will be confirmed using loss-of-function approaches.
